# Intracameral Triamcinolone Acetonide Versus Topical Dexamethasone: A Comparison of Anti-inflammatory Effects After Phacoemulsification

**DOI:** 10.7759/cureus.7592

**Published:** 2020-04-08

**Authors:** Kamran Haider Shaheen, Muhammad Saad Ullah, Syed Ahmer Hussain, Aamir Furqan

**Affiliations:** 1 Ophthalmology, District Headquarter Teaching Hospital, Dera Ghazi Khan, PAK; 2 Anesthesia and Critical Care, Chaudhry Pervaiz Elahi Institute of Cardiology, Multan, PAK

**Keywords:** cataract, inflammation, phacoemulsification, triamcinolone

## Abstract

Study objective and design

The objective of this study was to determine the effectiveness of triamcinolone acetonide when used as a single dose as compared to the topical use of dexamethasone to control the inflammation after phacoemulsification. The study was a randomized controlled trial conducted in the Department of Ophthalmology at the District Headquarter (DHQ) Teaching Hospital, Dera Ghazi Khan, from March 1, 2018, to August 31, 2019.

Materials and methods

Eighty patients were included in the study. All patients were assigned to two groups of 40 patients each using the lottery method. Group A patients were treated with a 1-mg intracameral injection of triamcinolone acetonide postoperatively after phacoemulsification. Group B patients were administered 0.1% dexamethasone eye drops with a dosage of one drop every four hours for four weeks. Postoperative follow-up was planned for day one, day seven, and day 28.

Results

The postoperative inflammation cell values of Group A on day one, day seven, and day 28 were 1.68 ±0.84, 0.22 ±0.15, and 0.12 ±0.23, respectively, while the postoperative inflammation cell values of Group B on day one, day seven, and day 28 were 1.91 ±0.75, 0.28 ±0.15, and 0.09 ±0.20, respectively. The postoperative inflammation flare values of Group A on day one, day seven, and day 28 were 0.31 ±0.37, 0.03 ±0.44, and 0.00 ±0.22, respectively, while the postoperative inflammation flare values of Group B on day one, day seven, and day 28 were 0.25 ±0.26, 0.22 ±0.46, and 0.02 ±0.18, respectively.

Conclusion

The efficacy of both modes of treatments is comparable; however, triamcinolone acetonide is preferable to dexamethasone, as its intracameral injection generally results in better compliance than multiple dosages of topical eye drops of dexamethasone.

## Introduction

Cataract is the most common cause of treatable blindness worldwide, with approximately 51.5% of blindness cases attributed to cataract [1,2]. People who are ≥50 years old are most commonly affected by cataracts, resulting in visual impairment (i.e., almost 65% worldwide) [3]. With the recent advancements in medical care and living standards throughout the world, the average age of the population is increasing along with the total population, which ultimately could result in an increased incidence of cataract cases. In both developing and developed countries, cataract is the leading cause of vision loss [4]. Similarly, cataract surgery is the most common procedure performed by ophthalmologists all over the world [5]. According to research, nearly 18 million cataract surgeries are performed by ophthalmologists to treat cataracts every year, and this number is on the rise along with the increasing population and average age of the population in the world [6].

The gold standard of treatment and the most commonly performed surgery for cataracts is small incision surgery with phacoemulsification [7]. However, postoperative inflammation can result in complications such as raised intraocular pressure (IOP), cystoid macular edema, increased recovery time, or formation of synechiae [8,9]. The most common treatment option for postoperative inflammation is topical steroids. Apart from the topical route, other means of administration of steroids include intravitreal, intracameral, subtenon, and subconjunctival [10]. Triamcinolone, when used via intracameral route, has a 100% effect after four weeks of phacoemulsification and is completely safe for treating postoperative inflammation after cataract surgery [11].

Waseem et al. conducted a study in Pakistan on the use of topical 0.1% dexamethasone, and the results showed that it was effective in 98% of cases after five weeks of phacoemulsification [12]. We are performing this study to compare the overall efficacy and safety of intracameral triamcinolone used as a single dose with topical use of dexamethasone for the treatment of postoperative inflammation in the local population. We believe this study will enable us to provide recommendations regarding the use of a more efficacious treatment option for the reduction of postoperative inflammation after phacoemulsification.

## Materials and methods

This randomized controlled trial was conducted in the Department of Ophthalmology at the District Headquarter (DHQ) Teaching Hospital, Dera Ghazi Khan, from March 1, 2018, to August 31, 2019. Approval for the study was obtained from the hospital’s ethics committee. The study conducted by Manzoor et al. was used to calculate the sample size [13]. Eighty patients were included in the study. Inclusion criteria included patients of either gender between the ages of 40 to 70 years who were admitted to the Department of Ophthalmology for phacoemulsification for cataract. Patients with any ocular pathology, anterior uveitis, or any intraoperative complication during surgery such as vitreous loss or posterior capsular rupture, history of prior surgery, and other comorbid conditions were excluded from the study.

Phacoemulsification with foldable lens implantation was performed under local anesthesia. All procedures were performed by a consultant surgeon with at least five years of experience. Group A patients were treated with 1-mg intracameral injection of triamcinolone acetonide postoperatively after phacoemulsification. Group B patients were administered 0.1% dexamethasone eye drops with a dosage of one drop every four hours for four weeks. All 80 patients were given 0.5% moxifloxacin eye drops at the rate of one drop every six hours for four weeks. Postoperative follow-up was planned for day one, day seven, and day 28. Inflammation was assessed based on cells in the anterior chamber and aqueous flare. Grading for cells in the anterior chamber was done as follows: grade 0=50. Similarly, aqueous flare grading was done as follows: 0=none, 1=just detectable or mild, 2=iris details clear or moderate, 3=iris details hazy or marked flare, 4=severe flare or heavy clots and fibrin deposits. IBM SPSS Statistics for Windows, Version 23.0 (IBM Corp., Armonk, NY) was used to perform the statistical analysis. Means and standard deviation were calculated for quantitative variables such as age, while frequency and percentage were calculated for qualitative variables such as gender. A Chi-square test was applied to compare the two groups in terms of efficacy. A p-value of ≤0.05 was considered statistically significant.

## Results

Eighty patients of both genders were included in this study. The mean age of Group A was 50 ±5 years. There were more male patients (n=22; 55%) than female patients (n=18; 45%). The mean age of patients in Group B was 51 ±4 years. In group B also, there were more male patients (n=29; 72.5%) than female patients (n=11; 27.5%). The differences were statistically insignificant (Table 1).

**Table 1:** Demographic characteristics of both groups

Variable	Group A (n=40)	Group B (n=40)	P-value
Mean age (years)	50 ±5	51 ±4	0.362
Gender			
Male	55% (n=22)	72.5% (n=29)	0.068
Female	45% (n=18)	27.5% (n=11)	

The postoperative inflammation cell values of Group A at day one, day seven, and day 28 were 1.68 ±0.84, 0.22 ±0.15, and 0.12 ±0.23, respectively, while the postoperative inflammation cell values of Group B at day one, day seven, and day 28 were 1.91 ±0.75, 0.28 ±0.15, and 0.09 ±0.20, respectively. The postoperative inflammation flare values of Group A at day one, day seven, and day 28 were 0.31 ±0.37, 0.03 ±0.44, and 0.00 ±0.22, respectively, while the postoperative inflammation flare values of Group B at day one, day seven, and day 28 were 0.25 ±0.26, 0.22 ±0.46, and 0.02 ±0.18, respectively. The differences were statistically insignificant (Table 2).

**Table 2:** Postoperative outcomes in both groups

		Group A (n=40)	Group B (n=40)	P-value
Cells	Day 1	1.68 ±0.84	1.91 ±0.75	0.094
	Day 7	0.22 ±0.15	0.28 ±0.15	0.123
	Day 28	0.12 ±0.23	0.09 ±0.20	0.662
Flare	Day 1	0.31 ±0.37	0.25 ±0.26	0.427
	Day 7	0.03 ±0.44	0.22 ±0.46	0.084
	Day 28	0.00 ±0.22	0.02 ±0.18	0.685

The mean IOP values of Group A at preoperative, day one, day seven, and day 28 were 17.14 ±2.17 mmHg, 17.78 ±2.75 mmHg, 15.54 ±2.46 mmHg, and 13.44 ±1.52 mmHg, respectively, while the mean IOP values of Group B at preoperative, day one, day seven, and day 28 were 16.02 ±2.55 mmHg, 17.43 ±3.36 mmHg, 15.41 ±2.21 mmHg, and 13.21 ±1.65 mmHg, respectively. The differences were statistically insignificant (Table 3).

**Table 3:** Comparison of intraocular pressure between groups

Mean intraocular pressure	Group A, mmHg (n=40)	Group B, mmHg (n=40)	P-value
Preoperative	17.14 ±2.17	16.02 ±2.55	0.069
Day 1	17.78 ±2.75	17.43 ±3.36	0.617
Day 7	15.54 ±2.46	15.41 ±2.21	0.783
Day 28	13.44 ±1.52	13.21 ±1.65	0.528

## Discussion

The treatment of postoperative complications of cataract surgery, especially inflammation, is important [13]. The use of steroids in the management of inflammation has been commonplace for a long time. The mechanism of the anti-inflammatory effect of steroids is their action on multiple intercellular mediators of inflammation. Steroids act by controlling the leakage of cells of inflammation and inhibit the formation of granulation tissue and the propagation of fibroblasts [14]. Intraocular injections of triamcinolone acetonide are commonly used in order to manage inflammatory diseases of the posterior chamber. In an experimental study conducted by Oh et al., they administered triamcinolone acetonide into the anterior chamber of a rabbit’s eyes to see its effect on the corneal epithelium [15]. The results of their study revealed no statistically significant decrease in central corneal thickness or any difference in endothelial cell count, but they did notice a reduced amount of microvilli.

Another study conducted by Chang et al. showed toxic effects with the use of triamcinolone acetonide on a cultured epithelium [16]. Even though there are reports of potential adverse effects of triamcinolone acetonide injections on the epithelium of the cornea, it is widely used currently in the treatment of postoperative inflammation after cataract surgery. Gills et al. conducted a study on the use of triamcinolone acetonide injection but could not find the optimum dosage for its use [17]. Another study was conducted where 60 patients were divided into two equal groups [18]. The intracameral triamcinolone group was administered the intracameral injection of triamcinolone along with gentamicin followed by eye drops of topical tobramycin at the rate of four drops daily for one week. In the other group (the topical group), dexamethasone was administered in combination with tobramycin in the form of topical eye drops after cataract surgery four times a day until the inflammation was healed. The results of that study showed no statistically significant difference between the two groups in terms of cell count in the anterior chamber after one week of phacoemulsification; however, the cell count difference in the anterior chamber was statistically significant one month after surgery, as cell count was lower in the intracameral triamcinolone group as compared to the topical group (P=0.006).

Intravitreal and intracameral triamcinolone has also been used in combination with topical steroid eye drops to treat postoperative inflammation after performing phacoemulsification in uveitic eyes, and it has proven to be effective [19,20]. The topical use of steroids has certain drawbacks, such as high cost, poor compliance from the patient, corneal irritation, and tear film disruption leading to irritation in the eyes. Therefore, in order to reduce these drawbacks of topical steroids, alternative methods of administration are being tried [21,22]. Triamcinolone acetonide intracameral injection is very efficacious in diminishing the inflammation that occurs after phacoemulsification, as reported by Karalezli et al. [23].

This study has some limitations. Our sample size was relatively small, as it involved only 80 patients in total, divided into two groups of 40 each. Further studies involving larger samples are recommended to be able to develop definitive guidelines.

## Conclusions

We found that the efficacy of both modes of treatments is comparable; however, triamcinolone acetonide is preferable to dexamethasone, as its intracameral injection generally results in better compliance than multiple dosages of topical eye drops of dexamethasone.
